# Effects of spermine NONOate and ATP on protein aggregation: light scattering evidences

**DOI:** 10.1186/2046-1682-6-1

**Published:** 2013-01-04

**Authors:** Rasha Bassam, Ilya Digel, Juergen Hescheler, Ayseguel Temiz Artmann, Gerhard M Artmann

**Affiliations:** 1 Institute of Bioengineering (IFB), Aachen University of Applied Sciences, 52428 Juelich, Germany; 2 Institute of Neurophysiology, University of Cologne, 50931 Cologne, Germany

## Abstract

**Background and objective:**

Regulating protein function in the cell by small molecules, provide a rapid, reversible and tunable tool of metabolic control. However, due to its complexity the issue is poorly studied so far. The effects of small solutes on protein behavior can be studied by examining changes of protein secondary structure, in its hydrodynamic radius as well as its thermal aggregation. The study aim was to investigate effects of adenosine-5’-triphosphate (ATP), spermine NONOate (NO donor) as well as sodium/potassium ions on thermal aggregation of albumin and hemoglobin. To follow aggregation of the proteins, their diffusion coefficients were measured by quasi-elastic light scattering (QELS) at constant pH (7.4) in the presence of solutes over a temperature range from 25°C to 80°C.

**Results and discussion:**

1) Spermine NONOate persistently decreased the hemoglobin aggregation temperature T_*a*_irrespectively of the Na^+^/K^+^ environment, 2) ATP alone had no effect on the protein’s thermal stability but it facilitated protein’s destabilization in the presence of spermine NONOate and 3) mutual effects of ATP and NO were strongly influenced by particular buffer ionic compositions.

**Conclusion:**

The ATP effect on protein aggregation was ambiguous: ATP alone had no effect on the protein’s thermal stability but it facilitated protein’s destabilization in the presence of nitric oxide. The magnitude and direction of the observed effects strongly depended on concentrations of K^+^ and Na^+^ in the solution.

## Background

Evidences that proteins significantly alter their conformation during a functioning cycle are numerous [1,2]. In spite of great success made by bioinformatics and structural biology, the particular mechanisms governing structural transitions remain obscure [1,2]. Revealing the principles of protein functional dynamics would be a key to understand many biological processes at the molecular level. The dynamic nature of protein functioning requires obtaining a multi-dimensional picture of each gene product, which means the one including not only its chemical composition and 3D structure but also its motional characteristics over time and temperature ranges. This is an extremely challenging task and one step towards its implementation can be made through protein denaturation studies. Protein denaturation is itself a very complex process, consisting of many molecular events, including unfolding, re-folding, aggregation, etc. [2]. Before [3-6] we reported for the first time that temperature-driven structural transitions in mammalian hemoglobin and other proteins are closely related to the corresponding body temperature and are genetically determined. Those data were obtained using micropipette aspiration technique [5], CD spectroscopy [4], light scattering [3] as well as NMR spectroscopy and colloid-osmotic pressure measurements [7]. The discovered phenomenon has been recently confirmed by neutron scattering studies [8,9]. We also addressed the modulating role of the protein environment, especially pH and Ca^2+^ concentration, in the manifestation of the phenomenon of hemoglobin’s structure transition at body temperature [10]. As a further logical development of those studies we have focused our interest now on the effects of common biological regulators such as nitric oxide and ATP on conformational and hydrodynamical properties of hemoglobin and serum albumin in various ionic environments. Recently, we have reported strong effects of ATP and spermine NONOate on hemoglobin’s secondary structure [11]. Without any doubt, protein conformational properties depend on multiple physico-chemical factors, such as temperature, pH, ionic strength as well as presence of numerous protein-binding groups and molecules. Studies on extremophilic organisms have revealed a vast spectrum of molecules that are able (and were indeed successfully used) to modify protein thermal stability. Many organic solutes, such as glycerol [12], 2-3- diphosphoglycerate (2,3 DPG) taurine [12], strombine and others, together with keeping the osmotic balance, may play an important role in stabilizing proteins in the face of thermal stress, protecting proteins from both heat and cold denaturation [12]. Thus, regulation of protein stability in the cell using small organic and inorganic molecules has evolutionary proved out as a rapid, reversible, and tunable method of metabolic control but its functioning in non-extremophilic organisms has been poorly studied so far. Among numerous low-molecular mediators of cellular activity, NO and ATP have attracted big interest, because of their ubiquity in living systems. Nitric oxide (NO) have long been known to play important role in physiology, pathology and pharmacology [13], being involved in numerous biological processes, such as vasodilatation [14], inhibition of platelet aggregation [15], blood pressure regulation [13], immune response [16], etc. Evidence is growing concerning multiple chemical mechanisms of NO interaction with proteins. They mostly involve the cysteine residues in proteins [17]. A process of introduction of nitric group into a protein molecule, known as nitrosylation, appears to be an important mechanism of cellular metabolic regulation but its implication in protein dynamics is still unclear [17]. Another omnipresent cellular messenger, adenosine 5’-triphosphate (ATP) can exert most of its actions by interacting with proteins, both inside and outside the cell [18]. Usually, proteins bind ATP by characteristic domains like the Rossmann fold [19] but, intriguingly, many observations suggest that even the proteins presumably not reacting with ATP can change their properties in presence of ATP. For example, the change in the concentration of ATP in the red blood cells (RBCs) results in alterations in Hb oxygen affinity [20]. In the red blood cells, the concentration of ATP appreciable (0.2 - 2.0 mM) and therefore can influence the properties of hemoglobin [20]. Considering protein stability, the role of ionic environment is also important for protein function. Even simple and ”trivial” cations like Na^+^ (native ionic radius 0.95 *Å*) and K^+^ (native ionic radius 1.33 *Å*) demonstrate complicated hydration behavior and influence the hydrogen bond network of water in a distinctly different ways [21]. Charged and polar groups of a protein interact with these ions in quite different manners. Potassium generally exhibits stronger affinity to surfaces of proteins as compared to sodium [21,22] that possibly contributes to K^+^/Na^+^-distribution inside/outside the cell [18,23]. In this work we focused mainly on aggregation aspects of protein denaturation. The aim of this study was to investigate the influence of ATP, NO donor as well as sodium/potassium balance on protein thermal stability using bovine serum albumin and human hemoglobin. Both proteins have been chosen because they are abundant in blood, their thermal behavior is well-studied (also by our group) and because of simplicity of their handling and purification.

## Methodology

### Buffers

In order to examine the role of K^+^/Na^+^balance in the medium in the NO- and ATP-induced effects, two different buffers were used for sample preparation: the sodium based phosphate buffered saline (PBS) (137 mM NaCl, 2.7 mM KCl, 8.1 mM Na_2_HPO_4_, 1.76 mM NaH_2_PO_4_) and its potassium-based analog buffer (referred later as CD-buffer) composed of 0.1 M KCl, 61.3 mM K_2_HPO_4_ and 5.33 mM KH_2_PO_4_. Both buffers had pH 7.4 and osmolarity 390 ± 10 mosm/l.

### Sample preparation

Human hemoglobin (Hb) was prepared directly from erythrocytes [6]. In brief, 50 *μ*L of heparinized blood were collected from donor’s fingertip, diluted by 1 mL of the Na- or K-buffer, correspondingly, and centrifuged at 800 g for 5 min. The supernatant was discarded and the RBC pellet was washed once again using the same procedure. The washed RBC pellet was hemolysed by adding 200 *μ*L distilled water. Afterwards, ionic strength and pH of the obtained hemoglobin solutions were adjusted by one of the buffers to mimic their natural values in blood [6]. Finally, the samples were centrifuged at 15000 g for 5 min to sediment cell membranes and the supernatant (Hb-solution) was filtered twice through a 0.2 *μ*m Whatman nitrocellulose filter and diluted by an appropriate buffer to obtain the standard QELS working concentration (1.0 mg/mL) and volume (6 mL) in the QELS scintillation vial. Hb concentration was determined according to [24] at 405 and 540 nm using V-550 spectrophotometer (Jasco Labor- und Datentechnik GmbH, Gross-Umstadt, Germany) using 1 cm thick quartz cuvettes (Hellma GmbH Co., KG, Muellheim, Germany). Hemoglobin prepared with this method is considered to be about 99% pure [3,25]. Bovine serum albumin, (BSA) ”Fraction V” was purchased from Sigma-Aldrich Co. (Munich, Germany). The working BSA solution (1 mg/mL) was prepared using an appropriate buffer in a dust-free environment. For the measurement, 6 mL of the working solution were filtered through 0.2 *μ*m Whatman nitrocellulose membrane filter (Sigma-Aldrich, Munich, Germany) into a QELS glass scintillation vial (Wheaton, USA) using a one-way plastic syringe. The pH value 7.4 ± 0.2, osmolarity and protein concentrations in the samples have been kept constant throughout all measurements.

### Sample treatment with nitric oxide and adenosine 5’-triphosphate

In this study a nitric oxide donor (spermine NONOate) was chosen for simplicity in its handling, storage stability and its convenient half-life in solution (about 39 min at 37°C) [13,14]. Spermine NONOate (N-(2-Aminoethyl)-N-(2-hydroxy-2-nitrosohydrazino)-1,2-ethylene-diamine) of 98% purity was purchased from MerckⒸ KGaA, Darmstadt, Germany. The use of NO donors also allows avoiding many difficulties inherent in gaseous nitric oxide (II) applications [14]. Nucleophilic complexes of NO with amines (spermine NONOates) appear to meet most of research criteria [26]. However, these compounds are self-decomposing in solution producing 2 mole of NO per mole of the substrate. Adenosine-5-triphosphate (di-sodium salt) of 98% purity was purchased from Carl Roth GmbH, Karlsruhe, Germany. Just before the measurement, freshly prepared spermine NONOate stock solution or ATP stock solution in the Na- or K-buffer was injected through a 0.2 *μ*m Whatman filter into the scintillation vial containing the sample to achieve the working concentration corresponding to 1:1 molecular ratio with the protein. In the control group measurement the same volume of corresponding pure buffer was added. Similarly, freshly prepared ATP stock solution was introduced into the sample to achieve the working concentration of 0.08 mg/mL (corresponding to 1:1 molecular ratio) in the scintillation vial immediately before the measurement. Both spermine NONOate and adenosine 5’-triphosphate are commercially acquired (Sigma-Aldrich Co., Munich, Germany).

### QELS measurement

The thermal stability of the proteins was defined by the temperature T_*a*_corresponding to the onset of the protein aggregation at a given rate of heating. The aggregation level was evaluated by quasi-elastic light scattering (QELS), a method, knowingly well-suited for denaturation studies, since the diffusion coefficients, measured by QELS, are determined by particle size, shape, and flexibility, as well as by inter-particle interactions. These parameters provide important information about the kinetics and structural transitions within systems of particles in solution [27]. QELS measurements were carried out using a Dawn^*®*^ EOSTM device equipped with a QELS module (both: Wyatt Technology Co., Santa Barbara, CA, USA) running in the batch mode. For data acquisition and analysis Astra^*®*^5.2 software was used. Initial temperature of the sample was adjusted to 25 0.1°C. During each measurement, the temperature was gradually increased up to 80°C at a rate of 0.5°C/min so that the usual measurement duration was about 110 min. These settings were kept consistent throughout the whole study [27]. From the diffusion coefficients (D) directly obtained from the QELS measurement, hydrodynamic radii (Rh) of the proteins were derived using the Astra software. Rh is the effective radius of a molecule in a solution and can be considered as the radius of a hard sphere that diffuses as the same rate as the molecule [27].

### Data analysis

Each experimental variant was measured independently at least in triplicate. The raw QELS data (diffusion coefficients and the hydrodynamic radii as functions of time as well as the denaturation temperature as a function of time) were initially extracted as ASCII text format. Typically, around 1500 Rh values were collected in a single experimental run. The obtained text data were initially processed by the MATLAB^*®*^software (Math Works, Massachusetts, USA) and exported into the Origin Pro 8^*®*^(Origin Lab Corporation, USA). The smoothing percentile algorithm was applied to exclude the artifacts produced due to dust particles and other noise types. This algorithm essentially performs a local polynomial regression to determine the smoothed value for each data point. The denaturation onset points were determined by measuring the slope of the experimental curves. When the hydrodynamic radius (nm) were plotted versus temperature, two distinct kink points usually appeared so that the curves had a characteristic l-shape (with increasing temperature: 1st gentle slope, steep slope). The aggregation temperature, T_*a*_, was calculated as the intersection point between the best-t tangential lines. To obtain comparisons between groups, mean values and corresponding standard errors were calculated [11].

## Results

### Spermine NONOate effects on Hb and BSA thermal aggregation

**Figure 1:**
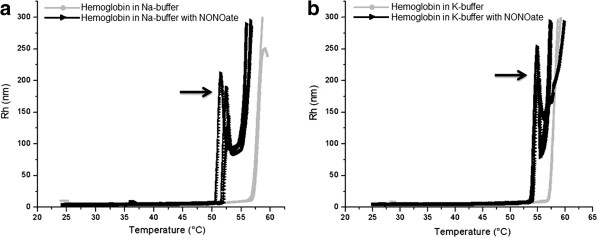
**Influence of NO on denaturation onset of Hb samples.** Influence of NO on denaturation onset of Hb samples (3 in each group) prepared in Na buffer (**a**) and the K buffer (**b**). Arrows indicate the appearance of an additional peak in the NO-treated samples that generally denatured significantly earlier than the control samples. In the region between 25°C to 45°C the Rh values for hemoglobin were around 5 nm in the Na buffer and around 4 nm in the K buffer, depending on the sample. For the temperatures exceeding 65°C the exact Rh values could not be measured because of pronounced size heterogeneity of the formed aggregates.

**Figure 2:**
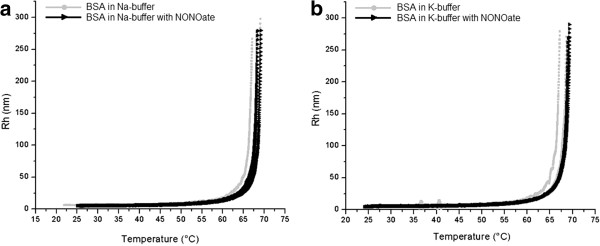
**Influence of NO on denaturation onset of BSA samples.** Influence of NO on denaturation onset of BSA samples (3 in each group) prepared in the Na buffer (**a**) and in the K buffer (**b**). In the region between 25°C to 45°C the Rh values for albumin were around 5.3 nm in both buffers. As the temperatures exceeded 70°C, the exact Rh values could not be measured anymore because of pronounced size heterogeneity of the formed aggregates.

For Hb samples prepared in Na buffer alone, the aggregation temperature (T_*a*_) was found to lie between 56.0°C and 57.0°C, slightly varying from sample to sample. Addition of spermine NONOate (114 M) to Hb samples prepared in Na buffer caused significant decrease in the T_*a*_, resulting in onset of denaturation between 50.0°C and 51.0°C (Figure 1a). Furthermore, in case of using the K buffer instead of the Na buffer, initial T_*a*_of Hb samples was between 56.0°C and 57.0°C, whereas the addition of the NO donor resulted in a shift of the T_*a*_towards 54.0°C (Figure 1b). Thus, for hemoglobin the increase in relative concentration of sodium in the medium (Na buffer) resulted in noticeable decrease of the protein thermal stability in the presence of the NO donor. Interestingly, for the entire NO donor-containing samples, an additional peak (indicated by arrows in Figure 1a, b) occurred. Such a peak has been never observed in the control (pure buffer) groups. The Rh values for Hb (control and NO donor treated samples) were around 5.0 nm in the Na buffer and around 4.0 nm in the K buffer, depending on the sample. For the temperatures exceeding 65.0°C the exact Rh values exceed 300.0 nm and could not be measured because of pronounced size heterogeneity of the formed aggregates and denaturated protein molecules. For the albumin samples prepared in pure Na buffer, the increase in hydrodynamic radii referred to denaturation occurred more gradually and within a broader temperature range (between 60.0°C and 65.0°C) as compared to Hb. In the case of K buffer, visible aggregation appeared within this range too (Figure 2a, b). Addition of NO donor somewhat reduces the denaturation temperature of both buffers (59.0°C to 61.0°C) but not significantly. In the region between 25.0°C to 45.0°C the Rh values for albumin were around 5.3 nm in both buffers. As the temperatures exceeded 70.0°C, the exact Rh values start to increase to 300.0 nm and also could not be measured anymore because of pronounced size heterogeneity of the formed aggregates.

### ATP effects on Hb and BSA thermal aggregation

**Figure 3:**
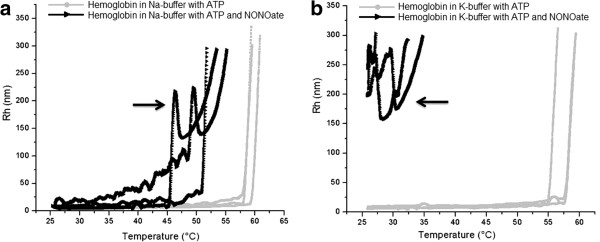
**Influence of ATP and NO on denaturation onset of Hb samples.** Influence of ATP and NO on denaturation onset of Hb samples (3 in each group) prepared in Na buffer (**a**) and K buffer (**b**). Arrows indicate the appearance of an additional peak in the NO-containing samples that denaturated markedly earlier than those without nitric oxide. The K buffer, containing both ATP and NO yet at room temperature (25°C) the average hydrodynamic radii reached values was 25 nm suggesting facilitated aggregation. Further increase in temperature induced formation of multiple large aggregates so that the measurement of Rh became impossible.

**Figure 4:**
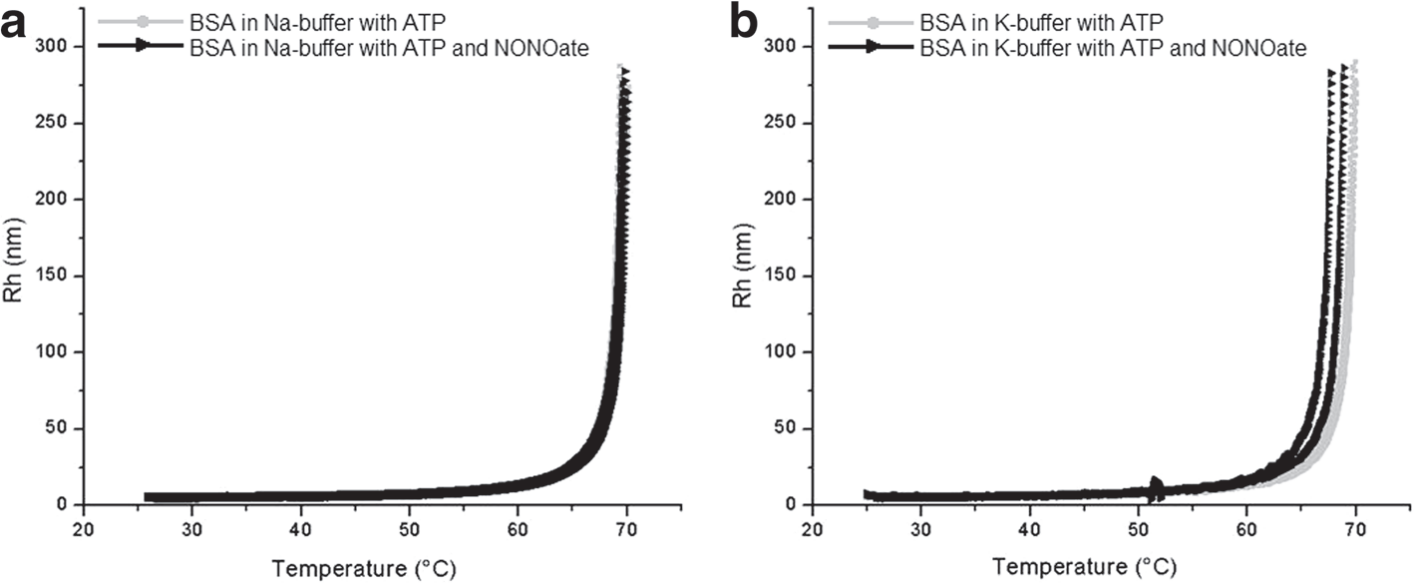
**Influence of ATP and NO on denaturation onset of BSA samples.** Influence of ATP and NO on denaturation onset of BSA samples (3 in each group) prepared in Na buffer (**a**) and K buffer (**b**). Control samples are marked with gray squares and NO treated samples are marked with black triangles. In the range of 25°C to 45°C the Rhs were around 5.1 nm and at a temperature above 70°C the Rhs could not be measured by the DLS due to its large size.

If only ATP was added to the Hb solution (Figure 3a, b, gray squares), protein aggregation began at 1-2°C higher temperature compared to control samples (Figure 1 and Figure 2). Remarkably, when ATP and spermine NONOate appeared in the solution together, (Figure 3a, black triangles), this resulted in dramatic increase in hemoglobin aggregation, even at relatively low temperatures (30°C - 40°C). This effect was especially profound in the K-buffer, where aggregation started immediately after the NO donor introduction (Figure 3b). Such a difference in behavior implies a significant role of the ions’ nature in this process. Again, the appearance of additional peaks in the curves from the NO donor-containing samples should be noted. An identical series of experiments under same conditions but with using albumin instead of hemoglobin revealed just minor changes after the NO donor introduction (Figure 4a, b). The experimental and control curves of BSA aggregation in the Na-buffer almost coincided, with the initial T_*a*_of being close to 65.0°C. The samples prepared in the K-buffer containing ATP aggregated near 66.0°C whereas the combination of spermine NONOate and ATP slightly shifted the T_*a*_towards 65.0°C in the Na-buffer and 63.0°C in the K-buffer. Table 1 summarizes the effects of spermine NONOate and ATP in the Na- and K-buffers on Hb and BSA thermal aggregation.

**Table 1:** Effect of NONOate and ATP on mean values of aggregation temperature Ta and hydrodynamic radius Rh of Hb and BSA in different ionic environments

**Hemoglobin**				
**Sample**	**Na-Buffer**		**K-Buffer**	
	***T***_*a*_**[°C]**	***R***_***h***_**(*nm*)**	***T***_***a***_**[°C]**	***R***_***h***_**(*nm*)**
Control	56.5	10.6	56.5	11.0
NONOate	50.5	7.7	54.0	10.2
ATP	58.0	27.3	57.6	13.3
NONOate+ATP	45.0	18.9	27.1	223.9
**Serum Albumin**				
**Sample**	**Na-Buffer**		**K-Buffer**	
	***T***_*a*_**[°C]**	***R***_***h***_**(*nm*)**	***T***_***a***_**[°C]**	***R***_***h***_**(*nm*)**
Control	62.5	15.0	63.0	17.1
NONOate	60.0	15.1	59.5	14.1
ATP	65.0	27.4	65.0	26.0
NONOate+ATP	65.3	25.5	63.0	27.1

## Discussion

Protein aggregation plays an important role in the cellular biology and in many applications of protein science and medical engineering [28]. Despite its biological importance, little is known about the launching mechanisms and potential pathways involved in the formation of molecular aggregates [29]. In this work we examined aggregation and diffusional behavior of proteins when implementing a combination of compounds like NO, ATP and increasing temperature. Our data have sustained the point of view that the denaturation point of a protein cannot be determined absolutely but depends strongly on particular composition, including monovalent ions.

### Facilitation of protein aggregation by NO

In our experiments, the temperature of distinct aggregation of Hb samples prepared in sodium- and potassium-based buffers decreased by ≈ 10°C when nitric oxide donor was added. In case of albumin a temperature shift caused by nitric oxide was observed, although to a lesser degree. Thus, in our experiments, a new biophysical aspect of nitric oxide action has been discovered, related to facilitation of thermal aggregation of proteins. We suppose that the minuteness of the “destabilizing” effect of NO on albumin might be related to the albumin’s structure, since this is a monomeric protein containing no iron. Several possible mechanisms can be suggested, governing the effects of NO on protein thermal stability. First, many effects of NO might be possibly mediated via chemical modification of proteins (nitrosylation). The role of nitrosylation in regulating signal transduction has been largely overlooked until relatively recently [30]. This is because the production of the small, highly reactive NO molecule had been thought to lack the specificity and control observed in other post-translational modifications such as phosphorylation [30]. Another plausible mechanism of the observed NO action can be its entry into protein’s hydrophobic core. Much of the biologically relevant information is concentrated in the non-polar residues that form the protein’s hydrophobic core [31-33]. The reaction of NO with oxygen will be accelerated in hydrophobic regions, with various consequences for the cell resulting from nitrosative stress [30]. Finally, the action of nitric oxide directly on the protein’s hydration shell can be speculated, influencing the hydrogen bonds network of the vicinal water. The corresponding changes solvent-exposed structures could be responsible for the onset of aggregation. At further thermal aggregation stages, the transition is dominated by the formation of aggregates and unfolding of the buried structures [34-36].

### ATP and NO: a synergy in inducing protein aggregation

Addition of ATP in our experiments systematically resulted in a slight increase of the protein aggregation temperature by 1-2°C. Thus, the effects of ATP alone on the aggregation of the studied proteins in both sodium- and potassium-based buffers can be gingerly defined as “mild stabilization”. However, if ATP and nitric oxide were added simultaneously, very intense aggregation of hemoglobin was observed, whereas albumin solution did not show any visible response. We have so far no plausible model explaining a physico-chemical nature of the pronounced synergetic action of ATP and NO in case of hemoglobin.

### Role of K^+^ and Na^+^ in protein’s thermal stability

Protein behavior inevitably depends on its ionic environment and one of our goals was to examine the effect of Na^+^/K^+^buffer composition on thermal stability of the studied proteins. The works of G. Ling [22] and other groups suggested that sodium and potassium ions should have different affinity to proteins, especially to denatured ones [22], and hence result in different aggregation behavior [37]. Therefore, there was a certain expectation from our side to observe the differences between the effects of these cations upon Hb and BSA aggregation. Indeed, in the case of combined action of NO and ATP, presence of potassium caused hemoglobin molecules to aggregate even at room temperature whereas in Na buffer the aggregation temperature was 20-25°C higher. Also, when NO donor was applied alone, the magnitude of its effect on protein stability greatly depended on the particular ion composition of the buffer: in the K buffer Hb aggregated at somewhat higher temperature. Interestingly, albumin solutions were much less sensitive to the sodium and potassium effects. Here we speculate that this could be referred to the fact that in contrast to Hb, BSA does not have a heme and a sub-unite architecture, making this molecule less susceptible to the changes in its physico-chemical environment. Because both osmolarity and pH were kept constant in the whole set of experiments, we believe that the observed effects can be attributed specifically to the concentrations and combinations of the compounds used in this study.

## Conclusion

In this work we examined thermal aggregation and diffusional behavior of proteins when implementing an NO donor, ATP as well as combination of the compounds. Our data have sustained the point of view that the denaturation point of a protein cannot be determined absolutely but depends strongly on the protein’s environment, including the type of the present monovalent ion. Summarizing, the main obtained results were: 1) depression of the hemoglobin’s denaturation temperature by NO donors; 2) distinct influence of ATP on the NO-mediated effects and 3) importance of the cationic composition of the medium for manifestation of the ATP- and NO-induced effects.

## Competing interests

The authors have declared that no competing interest exists.

## Authors’ contributions

RB performed the experimental work and data analysis. In addition, she wrote the whole paper. ID supervised and coordinate the experimental work for this research project and reviewed the paper. JH reviewed and approved the paper. ATA reviewed and approved the paper. GMA reviewed, approved and supervise the whole research work. All authors read and approved the final manuscript.
